# Gal-3 Protein Expression and Localization in Prostate Tumours

**DOI:** 10.3390/curroncol30030206

**Published:** 2023-02-23

**Authors:** Tânia Lima, Catarina Macedo-Silva, Diana Felizardo, João Fraga, Isa Carneiro, Carmen Jerónimo, Rui Henrique, Margarida Fardilha, Rui Vitorino

**Affiliations:** 1 Department of Medical Sciences, Institute of Biomedicine-iBiMED, University of Aveiro, 3810-193 Aveiro, Portugal; 2 Cancer Biology and Epigenetics Group, IPO Porto Research Center (GEBC CI-IPOP), Portuguese Oncology Institute of Porto (IPO Porto), 4200-072 Porto, Portugal; 3 Porto Comprehensive Cancer Center (P.CCC), 4200-072 Porto, Portugal; 4 Department of Pathology, Portuguese Oncology Institute of Porto (IPO Porto), 4200-072 Porto, Portugal; 5 Department of Pathology and Molecular Immunology, School of Medicine and Biomedical Sciences, University of Porto (ICBAS-UP), 4050-513 Porto, Portugal; 6 UnIC@RISE, Department of Surgery and Physiology, Faculty of Medicine of the University of Porto, 4200-319 Porto, Portugal; 7 LAQV/REQUIMTE, Department of Chemistry, University of Aveiro, 3810-193 Aveiro, Portugal

**Keywords:** Galectin-3, prostate tissue, marker, prostate cancer, localization, patient survival

## Abstract

Gal-3 plays an important role in cell survival, mRNA splicing, and cell–cell and cell–matrix interactions. Depending on its cellular localization and cancer type, Gal-3 may have tumour-suppressive or tumour-promoting activities. Given the promising diagnostic role of Gal-3 in the urine of PCa patients found in our previous study, its concordant gene and protein expression levels, and its involvement in PCa-related biological processes (e.g., morphogenesis of the prostate gland epithelium), we aimed to investigate this protein immunohistochemically in tumour and normal prostate tissues. Gal-3 protein expression was evaluated in 48 tumour prostate tissues, eight normal prostate tissues and 14 adjacent-normal prostate tissues. Decreased Gal-3 staining was detected in tumour tissues compared with normal tissues. Although Gal-3 staining was decreased in tumour tissues with GS 5-8 and pT2 and pT3 stages compared with normal prostate tissue, no correlation was found between Gal-3 expression and PCa progression. In the present study, the pattern of cellular localization differed between groups, as Gal-3 was predominantly excluded from the nucleus in tumour tissues. Furthermore, Gal-3 had no significant effect on survival and relapse in these PCa patients. This work confirms Gal-3 as a promising marker for PCa diagnosis.

## 1. Introduction

Galectins are carbohydrate-binding proteins, with β-galactoside binding activity and conserved carbohydrate-recognition domains (CRDs). Galectins have a broad spectrum of biological activity, namely in inflammation, immune response, apoptosis, cell survival, growth, adhesion, and migration. To date, 15 galectins are known, numbered according to the date of discovery, and classified into three groups according to the organization of CRDs: the prototype group with only one CRD (Galectins-1, -2, -5, -7, -10, -13, -14, and -15), the tandem repeats group with two CRDs (Galectins-4, -6, -8, -9, and -12), and the chimera group with one CRD linked to an N-terminal domain (Galectin-3) [1]. Galectin-3 (Gal-3), encoded by the LGALS3 gene, contains an amino terminal domain (N-terminal domain) that modulates its nuclear–cytoplasmic shuttling [2] and secretion [3], a repeated proline-rich domain resembling α-collagen and susceptible to cleavage by proteases and phosphorylation on some residues [4,5], and a C-terminal domain containing a CRD with an anti-death NWGR motif [6]. Synthesized in the cytoplasm, Gal-3 can also be translocated to the nucleus or secreted extracellularly and into biological fluids (e.g., serum and urine) by a non-classical mechanism that still remains to be elucidated [7]. Intracellularly, Gal-3 controls apoptosis [6], AKT phosphorylation [8], cell cycle regulation [9], Wnt/β-catenin pathway [10], and pre-mRNA splicing [11], while extracellularly it regulates cell-to-cell [12] and cell-to-matrix interactions [13]. Gal-3 usually occurs as a monomer, but can associate with other molecules through its N-terminal domain and/or CRD to modulate its biological functions. Gal-3 can self-associate through intramolecular interactions between the N-terminal domain and the CRD [14], or through intermolecular interactions between the two N-terminal domains [15] and two CRDs [16], resulting in open or closed conformations, which affects its stability and activation. Gal-3 can also bind to glycoproteins and glycolipids, forming dynamic conformations called lattices that regulate the structural organization of these molecules and processes, such as immune response, metabolism, and regulation of receptor kinases [17]. The activity, localization and interaction of Gal-3 with ligands is also modulated by post-translational modifications, such as cleavage and phosphorylation. Gal-3 is cleaved by MMPs and kallikreins, and promotes the process of angiogenesis [18,19]. The tyrosine and serine residues of Gal-3 can be phosphorylated by several enzymes (e.g., casein kinase I, glycogen synthase kinase 3β) [20], and this process is required for the shuttling between the nucleus and the cytoplasm [21].

Gal-3 is a relatively well studied protein in cancer, because it is involved in tumour immunosuppression, apoptosis/survival of tumour cells and tumour metabolism [22]. Just as Gal-3 is differentially expressed by a variety of cells and tissues [23], its expression also varies depending on the type and stage of cancer. For example, some cancers show reduced expression of Gal-3 [24,25,26], while others show increased Gal-3 expression [27,28,29]. The cellular localization of Gal-3 also appears to be relevant and to vary according to the type of cancer [30,31]. In PCa, this protein has been less studied. Several studies have reported a decrease in Gal-3 expression in primary and metastatic prostate tumours compared to normal or pre-malignant tissues [25,32,33,34]. This loss of Gal-3 expression is also observed in hormone sensitive- compared to hormone-insensitive PCa [25], and in malignant prostate cells compared to normal prostate cells [35]. It has been suggested that Gal-3 downregulation may be due to its cleavage, for example, by PSA [36], or to hypermethylation of its gene promoter [35,37]. In addition to differential protein expression, the localization of Gal-3 also appears to change during malignant transformation in the prostate. In fact, a translocation of Gal-3 from the nucleus to the cytoplasm has been observed in tumour prostate tissue [30,37]. Depending on its localization in the nucleus or cytoplasm, Gal-3 may have antitumor or tumorigenic activities, respectively [30]. However, this change in the Gal-3 cellular localization has not been observed elsewhere [38].

Given the promising diagnostic role of Gal-3 in the urine of PCa patients found in our previous studies [39], its concordant gene and protein expression levels, and its involvement in PCa-related biological processes (e.g., prostate-gland epithelium morphogenesis, cell migration and invasion) [39], we aimed to study this protein immunohistochemically in prostate tissue.

## 2. Materials and Methods

### 2.1. Patients and Samples

Seventy prostate specimens were obtained from the archives of the Department of Pathology of the IPO Porto, Portugal, and classified according to the 2022 World Health Organization (WHO) Classification of Tumours of the Urinary System and Male Genital Organs [40]. The tissue slides contained normal prostate tissue from patients without PCa (tumour-free prostates) (*n* = 8), adjacent-normal prostate (only normal tissue from patients diagnosed with Pca) (*n* = 14) and tumour prostate tissues with GS = 5 (*n* = 10), GS = 6 (*n* = 9), GS = 7 (*n* = 16), GS = 8 (*n* = 7) and GS = 9 (*n* = 6). The slides with tumour prostate tissues included tumour cells and adjacent-normal cells surrounding the tumour, and therefore interacting directly with the tumour cells. The clinical data of the patients who participated in this study are presented in Table 1. This study was approved by the Institutional Review Board (Comissão de Ética para a Saúde) of the Portuguese Oncology Institute of Porto (CES.15/016).

### 2.2. Immunohistochemical Evaluation of Galectin-3 Expression and Localization in Normal and Tumour Prostate Tissue (In Vivo Analysis)

Gal-3 protein expression was evaluated in prostate tissues by IHC, using a NovoLinkTM Max Polymer Detection System (Leica Biosystems, Wetzlar, Germany). After deparaffinization and rehydration of tissue sections, antigen retrieval was performed in 1 × sodium citrate buffer solution, pH 6.0, using a microwave at 800 W for 20 min. A 3% hydrogen peroxide solution was used to block endogenous peroxidase activity for 10 min. Tissue slides were then blocked with horse serum (1:50 dilution) for 20 min and then incubated with primary Gal-3 antibody (1:250 dilution, sc-32790, Santa Cruz Biotechnology) for 1 h at room temperature. Tissue sections were then incubated with post-primary, followed by polymer for 30 min each. Then, 3,3′-diaminobenzidine (DAB) (Sigma-Aldrich™, Darmstadt, Germany) was used as a chromogen, and hematoxylin (Leica Biosystems, Germany) as a counterstain. Selection of positive controls relied on the analysis of the HPA database (https://www.proteinatlas.org/humanproteome/tissue, accessed on 7 March 2022) [41] and one positive control tissue was used in each IHC experiment.

Immunohistochemistry evaluation of Gal-3 expression was performed by two experienced pathologists. For each slide, the intensity, extension, and cellular localization were graded. The intensity of the staining was graded as the following: negative (0), weak (1), moderate (2), and strong (3), while the extension was classified as: no positive cells (0), 1–25% positive cells (1), 25–50% positive cells (2), 50–75% positive cells (3) and more than 75% of positive cells (4). Protein cellular localization was categorised as cytoplasmic, nuclear, or both. The IHC score was calculated as the sum of the intensity and extension scores.

### 2.3. Exploration of Galectin-3 Expression in PCa and Its Impact on Patient Survival in Public Cancer Databases (In Silico Analysis)

To compare the results of Gal-3 protein expression in our cohort with other public cancer databases, GEPIA and UALCAN web serves were used. GEPIA (Gene Expression Profiling Interactive Analysis) is a web server for analysing the RNA sequencing expression data of thousands of tumours and normal samples from the TCGA and the GTEx projects. Depending on the cancer type and/or disease stage, GEPIA offers differential expression, survival, and correlation analyses, among others [42]. UALCAN is a web tool that offers an easy access to publicly available cancer OMICS data (TCGA, MET500, CPTAC and CBTTC), allowing to explore, analyse and visualize cancer OMICS data. In this way, UALCAN enables the identification of biomarkers or validation of genes of interest, analysis of gene expression data and promoter methylation profiles, and Kaplan–Meier survival analysis. RNA-seq data from TCGA can be accessed and analysed for 33 different tumour types. For PCa, two datasets are available, namely prostate adenocarcinoma and metastatic prostate cancer (MET500 dataset). In the present study, the prostate adenocarcinoma dataset was explored to obtain information regarding LGALS3 expression and promoter methylation profile in normal and tumour prostate tissue, as well as the effects of LGALS3 expression on PCa patient survival [43]. Promoter methylation levels are indicated by a beta value ranging from 0 (unmethylated) to 1 (fully methylated). Hypermethylation and hypomethylation were defined as a beta value of 0.7–0.5 and beta value of 0.3–0.25, respectively. LGALS3 expression was compared between normal and tumour prostate tissue, and across tumours with different GS.

### 2.4. Statistical Analysis

Statistical analysis of Gal-3 IHC scores was performed using GraphPad Prism (version 6.0) (GraphPad Software, San Diego, CA, USA). Comparison of Gal-3 expression levels between groups was performed using Mann–Whitney, Wilcoxon matched-pairs signed rank test, or Kruskal–Wallis tests, and adjusted for multiple comparisons using a Dunn’s test. Correlations between Gal-3 levels and other variables were assessed using a Spearman’s nonparametric test. To assess differences in cellular localization of Gal-3 in different groups, cytoplasmic localization was defined as 1, whereas cytoplasmic and nuclear localization were defined as 2. To explore the impact of Gal-3 expression on PCa patient survival and relapse-free survival, Kaplan–Meier statistics were used, and the corresponding curves were plotted. Based on the frequency distribution of the data, tumour samples were classified into low or high Gal-3 expression. For this purpose, an IHC score of 2, corresponding to the 75th percentile, was defined as the cut-off for high or low expression.

## 3. Results

### 3.1. Immunohistochemical Evaluation of Galectin-3 Expression and Localization in Normal and Tumour Prostate Tissue (In Vivo Analysis)

**Figure 1:**
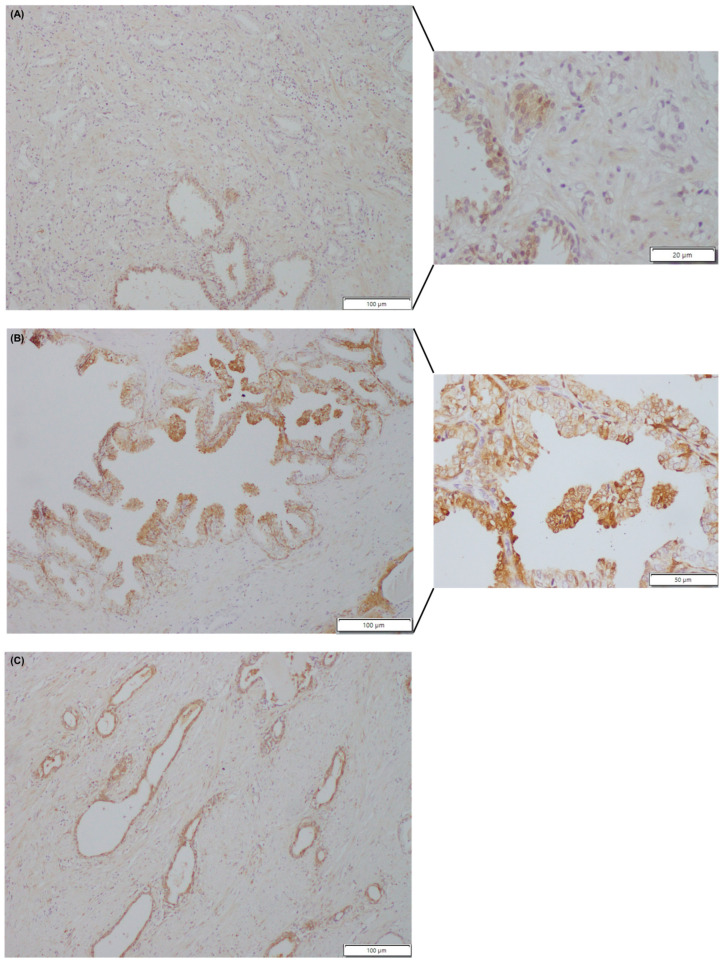
Gal-3 expression in prostate slides. (**A**) Tumour prostate tissue. Note: adenocarcinoma showing negative staining and non-neoplastic glands with moderate cytoplasmic and nuclear staining. (**B**) Adjacent-normal prostate tissue. Note: detail of non-neoplastic glands with nuclear membrane staining. (**C**) Non-neoplastic prostate. Atrophic glands showing moderate staining. Magnification factors of images A, B, C: ×10 and 40×; ×10 and 40×; 10×, respectively).

**Figure 2:**
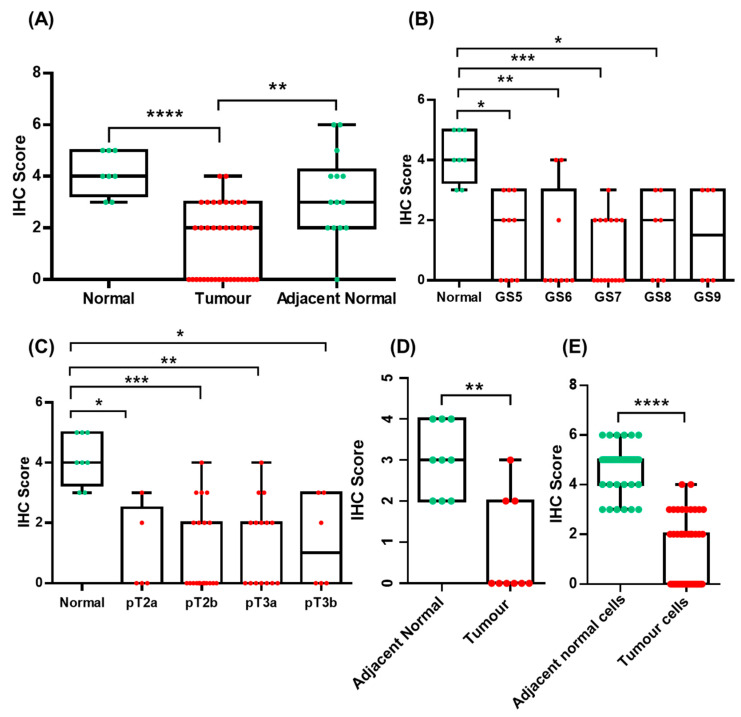
Gal-3 expression in normal, tumour and adjacent-normal prostate tissues (**A**), in normal and tumour prostate tissues with different GSs (**B**) and clinical stages (**C**). Gal-3 expression in tumour-matched adjacent-normal prostate and tumour prostate tissues (**D**). Gal-3 expression in co-localized adjacent-normal cells and tumour cells (**E**). (* *p* < 0.05; ** *p* < 0.01; *** *p* < 0.001; **** *p* < 0.0001).

**Figure 3:**
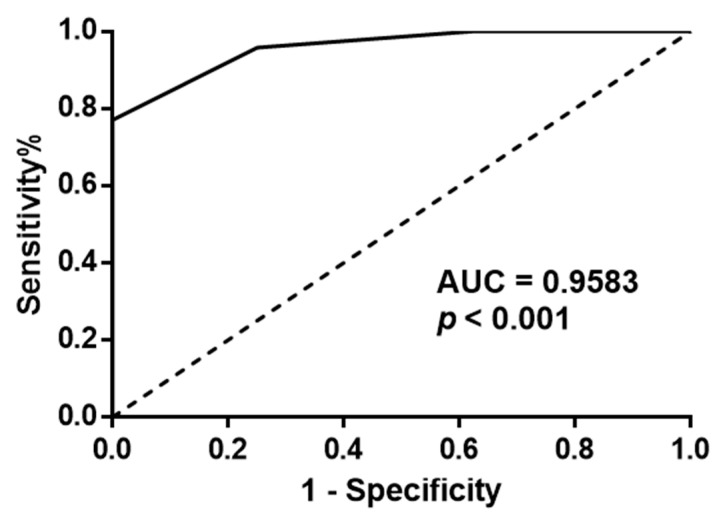
ROC curve of Gal-3 protein expression in normal and tumour prostate tissues. Area under the curve (AUC) = 0.9583(95% CI: 0.9042–1.012), *p*-value < 0.001.

No significant differences were found when comparing the age of the different study subjects (*p* = 0.5963). Gal-3 protein expression was significantly decreased in tumour prostate tissue compared to normal (*p* < 0.0001) and adjacent-normal prostate tissues (*p* = 0.0025), while no significant differences in Gal-3 expression were detected between normal and adjacent-normal prostate tissues (*p* = 0.5775) (Figure 1 and Figure 2A). Decreased Gal-3 protein expression in PCa specimens did not correlate with GS, stage, or serum PSA levels. Statistically significant differences were observed when comparing different GS groups (GS 5–9) and different clinical stages (pT2, pT3) with normal prostate tissue (Figure 2B,C). These differences were found between tumours with GS 5, 6, 7, and 8, and normal prostate tissue (*p* = 0.0256; *p* = 0.0047; *p* = 0.0003; *p* = 0.0458) (Figure 2B), and between pT2a, pT2b, pT3a and pT3b tumours, and normal prostate tissue (*p* = 0.0182; *p* = 0.0003; *p* = 0.0030; *p* = 0.0384) (Figure 2C). Nonetheless, no significant differences were observed between the groups of different GSs. Some adjacent-normal prostate tissues were tumour-matched (*n* = 9 cases). A significant decline in Gal-3 expression was observed in tumour tissues compared to the corresponding adjacent-normal prostate tissues (*p* = 0.0039) (Figure 2D). Comparison between tumour cells and co-localized adjacent-normal cells also revealed a significant decrease in Gal-3 protein levels in the former, following the trend of Gal-3 variation in tumour and normal tissues (*p* < 0.0001) (Figure 2E). To assess the usefulness of Gal-3 in discriminating between tumour and normal prostate tissues, a receiver operating characteristic (ROC) curve was performed using the Gal-3 protein expression levels in these samples. This ROC curve yielded an area under the curve (AUC) of 0.9583 (95% CI: 0.9042–1.012) and a *p*-value < 0.001, indicating that Gal-3 protein expression levels are useful in discriminating between tumour and normal prostate tissues (Figure 3).

**Figure 4:**
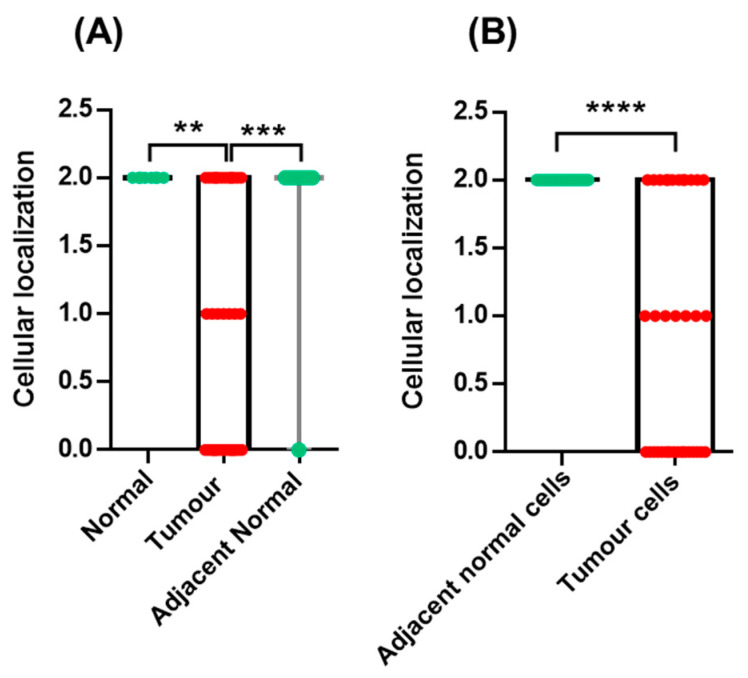
Gal-3 cellular localization in normal, tumour and adjacent-normal prostate tissues (**A**) and in co-localized adjacent-normal cells and tumour cells (**B**). The cytoplasmic location of Gal-3 is indicated by 1, while the cytoplasmic and nuclear location is indicated by 2. (** *p* < 0.01; *** *p* < 0.001; **** *p* < 0.0001).

In terms of cellular localization, Gal-3 showed both nuclear and cytoplasmic staining in normal, adjacent-normal and tumour tissues. Nevertheless, the pattern of cellular localization was different between groups, as cellular redistribution of Gal-3 towards the cytoplasm was observed in tumour cells. In fact, the presence of positively stained nuclei in normal (*p* = 0.0026) and adjacent-normal tissues (*p* < 0.0008) was significantly more evident than in tumour tissues, as well as in co-localized adjacent-normal cells (*p* < 0.0001) relative to tumour cells (Figure 4). In addition to prostate glandular cells, Gal-3 was also stained, in stromal cells in some cases.

### 3.2. Impact of Galectin-3 Expression on PCa Patient Survival and Relapse (In Vivo Analysis)

**Figure 5:**
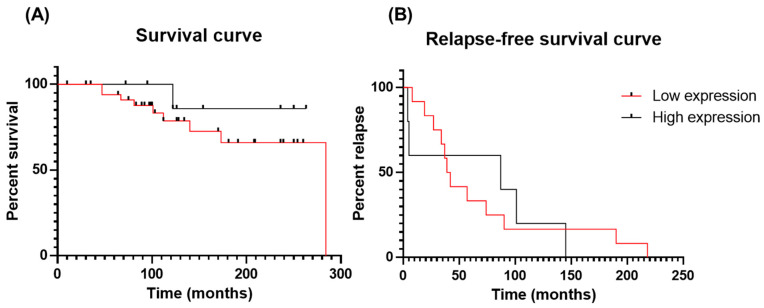
Impact of Gal-3 expression on PCa patient survival (**A**) (*p* = 0.36) and relapse-free survival (**B**) (*p* = 0.95).

Follow-up information was available for 46 of the 48 PCa patients (Table 1). Of these, three were deceased at the last follow-up, 10 died with the disease, five died without disease, two were alive, seven were alive with disease and 19 were alive without the disease. Of the 46 PCa patients, 17 patients experienced disease relapse, whereas 29 did not. One of the PCa patients with a GS of 7, and another with a GS of 8, suffered from liver and bone metastases, respectively. The survival and relapse data were used to plot the corresponding curves using the Kaplan–Meier statistics. This analysis revealed that Gal-3 protein levels had no significant impact on the survival or relapse of PCa patients (survival: *p* = 0.36; relapse: *p* = 0.95). The curves of survival and relapse-free survival are represented in Figure 5A,B, respectively.

### 3.3. Exploration of Galectin-3 Expression in PCa and Its Impact on Patient Survival in Public Cancer Databases (In Silico Analysis)

**Figure 6:**
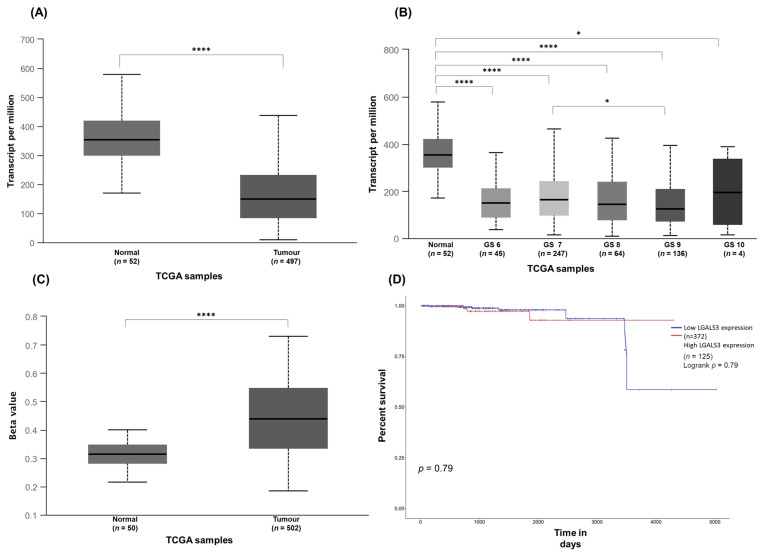
UALCAN analysis of: Gal-3 expression in normal and tumour prostate tissues from TCGA PCa cohorts (**A**), in normal and tumour prostate tissues with different GSs (**B**), Gal-3 promoter methylation levels in normal and tumour prostate tissues from TCGA PCa cohorts (**C**), impact of Gal-3 expression on PCa patient survival (**D**). (* *p* < 0.05; **** *p* < 0.0001).

**Figure 7:**
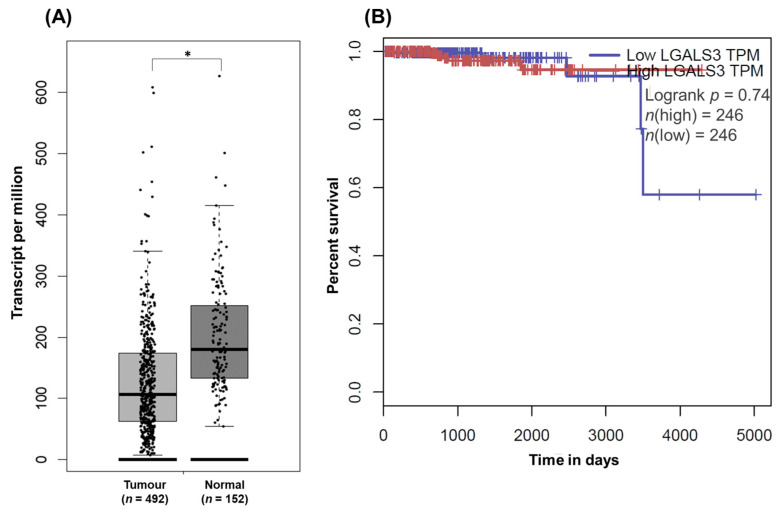
GEPIA analysis of Gal-3 expression in normal and tumour prostate tissues (**A**) and its impact on PCa patient survival (**B**). (* *p* < 0.05).

UALCAN and GEPIA were used to study the Gal-3 gene expression and its impact on the PCa patient survival. According to the results of the present work (Figure 2), in silico analysis revealed that the expression of Gal-3 was found to be decreased in tumours (*p* < 1 × 10^−12^) and prostate tumours with a GS of 6 (*p* = 1.63 × 10^−12^), 7 (*p* < 1 × 10^−12^), and 8 (*p* = 2.11 × 10^−12^), compared with normal prostate (Figure 6A,B and Figure 7A). Contrary to the results of the present work, the expression of Gal-3 was also significantly diminished in tumours with a GS of 9 (*p* = 1.62 × 10^−12^) compared with normal prostate (Figure 6B). To understand the likely events underlying the differential expression of Gal-3 in tumour and normal tissues, the Gal-3 promoter methylation profile was investigated in silico. The results showed that hypermethylation of the Gal-3 promoter could be one of the events underlying its downregulation in tumour tissue compared to normal tissue (*p* = 1.62 × 10^−12^) (Figure 6C). As for the effect of Gal-3 expression on the survival of PCa patients, both the results of this study (Figure 5), and those of the exploratory analysis using UALCAN and GEPIA, showed that this effect was not statistically significant (*p* = 0.79; *p* = 0.74) (Figure 6D and Figure 7B).

## 4. Discussion

The role of Gal-3 has been relatively well explored in various types of cancer, but little is known about its role in PCa (indicated by the paucity of publications on this topic). To the best of our knowledge, we were the first group to assess urinary Gal-3 levels in PCa patients and non-cancer subjects for diagnostic purposes. We found a significant downregulation of urinary Gal-3 levels in PCa patients, and therefore we wanted to investigate the expression of Gal-3 in tumour and normal prostate tissue [39]. 

Consistent with the results of other studies [25,30,32,33,34,37,38,44,45], this study found decreased Gal-3 staining in tumour prostate tissues compared with normal tissues. Despite the consensual downregulation of Gal-3 in PCa, the relationship between Gal-3 and disease progression has not been clearly established. On the one hand, the loss of Gal-3 appears to be associated with the progression of PCa, as it occurs in hormone-refractory disease relative to hormone-sensitive PCa, and correlates with tumour aggressiveness (GS and disease stage) [25,32,37]. On the other hand, no correlation was found between Gal-3 expression and the Gleason pattern or disease stage [30,38,44,45]. 

In the present study, Gal-3 staining in normal prostate was statistically different from that found in tumour tissues with GS 5,6,7,8, or with pT2 and pT3 stages, but did no correlate with the Gleason pattern or disease stage. Thus, Gal-3 staining does not appear to be associated with PCa progression in the present study. 

Following the same trend of variation in tumour and normal tissues, a decline in Gal-3 levels was observed in tumour tissues compared with their matched-adjacent-normal tissues, as well as in tumour cells compared with co-localized adjacent-normal cells. Interestingly, in some tumour prostate tissues, Gal-3 staining was detected in stromal cells in addition to the staining of prostate gland cells, which has not been reported in other studies [25,38].

In the present study, two types of tissues were used as controls: normal tissues patients diagnosed with PCa (adjacent-normal tissues) and normal tissues from patients without PCa (normal prostate tissue, tumour-free prostates). The Gal-3 staining pattern was different in these groups, although not significantly, as a strong staining pattern was observed in normal prostate tissues and an intermediate staining pattern was observed in adjacent-normal prostate tissues. This observation is consistent with other studies [25,38].

Both types of tissues have advantages and disadvantages that may affect study findings. On the one hand, adjacent-normal tissues minimize the confounding effects that may result from inter-individual variability; on the other hand, molecular alterations compatible with an intermediate, pre-neoplastic state have been detected in this tissue [46,47]. 

Indeed, Chandran and colleagues detected larger differential gene expression in tumour relative to normal prostate, than in tumour tissues compared with an adjacent-normal prostate. Moreover, upregulation of oncogenes, signal transducers, transcription factors and growth regulators was observed in adjacent-normal prostate compared with tumour-free prostate, resembling the tumour tissue profile [48]. Aran et al. observed that the transcriptome profile of adjacent-normal tissue in eight types of cancers, including PCa, represents an intermediate state between normal prostate and tumour. They examined and analysed transcriptome profiles of normal, adjacent-normal, and tumour tissues of various cancers from the TCGA and GTEx programs. Several genetic differences were found between normal and adjacent-normal tissues in all cancer types. Furthermore, a higher percentage of differentially expressed genes was found in tumour relative to normal tissue, than in tumour compared with adjacent-normal tissue. These authors therefore propose that the tumour modulates adjacent tissue and that tumour-induced changes in adjacent tissue may influence tumorigenesis and/or tumour progression [47].

Hypermethylation of the Gal-3 promoter [35,37] and Gal-3 cleavage by proteases, such as PSA [36,49], have been suggested as possible causes of the downregulation of Gal-3 in PCa. 

Accordingly, in silico analysis with UALCAN showed that the Gal-3 promoter methylation levels were increased in tumour compared to normal prostate tissues. Regarding the cleavage state of Gal-3, it was hypothesized that Gal-3 cleavage by PSA occurs during PCa progression, but not in normal or benign prostate tissues. Increased levels of cleaved Gal-3 and reduced levels of intact Gal-3 have been reported during PCa progression [38]. Both the intact and cleaved forms of Gal-3 have been claimed as diagnostic and therapeutic targets of PCa [36,49]. Nevertheless, Knapp and colleagues found no correlation between Gal-3 and PSA levels [38]. Corroborating the findings of Knapp and his team, we also found no association between Gal-3 and PSA levels in the present study. 

In addition to expression, the cellular localization of Gal-3 also appears to be an important factor in disease progression. It has been suggested that Gal-3 has opposite roles depending on its cellular localization, acting as a tumour suppressor in the nucleus and as a tumour promoter in the cytoplasm [50]. In the present study, the pattern of cellular localization was different among the groups. In tumour cells, Gal-3 was predominantly localized in the cytoplasm, in contrast to its nuclear and cytoplasmic localization in normal and adjacent-normal tissues. These findings are consistent with the tumour promoter function hypothesised for cytoplasmic Gal-3 [50]. Our results, like those of Van Den Brûle et al., point to an exclusion of Gal-3 from the nucleus to the cytoplasm [30], but contradict those of Knapp and team, who reported an identical pattern of Gal-3 cellular localization in normal and tumours prostate tissues [38].

Kaplan–Meier statistics were used to address the question of whether Gal-3 expression impacts the survival and relapse of PCa patients. Results showed that Gal-3 had no significant impact on the survival of these patients, which is consistent with the analysis of TCGA PCa cohorts using UALCAN and GEPIA. Gal-3 also had no significant impact on the relapse of PCa patients, which other studies did not confirm [30,38,51]. 

On the other hand, the ROC curve showed the diagnostic ability of Gal-3 to distinguish between tumour and normal prostate tissues. Thus, these findings highlight the diagnostic potential of Gal-3 in PCa, but it seems that Gal-3 is not a prognostic biomarker for patient survival. 

The mode of action of Gal-3 promoter hypermethylation in relation to survival or relapse of PCa patients is not fully understood. However, it is believed that the methylation of the Gal-3 promoter may impair the expression of Gal-3 protein in PCa cells, leading to increased tumorigenic activity. Furthermore, the inhibition of Gal-3 protein expression could reduce the tumour suppressor activity of Gal-3, resulting in a more aggressive cancer phenotype. Additionally, the reduced expression of Gal-3 protein in PCa cells could lead to a decreased antitumor immune response, which might contribute to the poor prognosis of PCa patients.

In this study, the aim was to investigate the differences in Gal-3 expression in tumour tissues, and not the mechanism of action behind it. However, it would be very interesting to evaluate Gal-3 promoter methylation in the tissue samples analysed in this study. As in PCa, different levels of Gal-3 methylation and expression have been found according to disease stage, with stages I and II showing greater methylation and a more pronounced decrease in Gal-3 expression than stages III and IV [37,44]. In our study, the decrease in Gal-3 expression was more pronounced in stage II than in stage III tumours. 

## 5. Conclusions

This work shows the diagnostic potential of Gal-3 in PCa. In view of these results, Gal-3 deserves to be further investigated in the context of PCa, especially its diagnostic role.

## Figures and Tables

**Table 1:** Clinical data of the subjects included in the study.

Characteristic	PCa *n* = 48	Normal Prostate *n* = 8	Adjacent-Normal Prostate*n* = 14
**Age (Years)**	64.10 ± 6.52	68.50 ± 16.72	65.14 ± 6.26
**Serum PSA (NG/ML)**	10.08 ± 4.54	_	9.64 ± 4.27
**Combined Gleason Score**	6.79 ± 1.29	_	7.21 ± 0.97
	GS5: 10 (20.83%) GS6: 9 (18.75%) GS7: 16 (33.33%) GS8: 7 (14.59%) GS9: 6 (12.5%)		GS6: 3 (21.43%) GS7: 7 (50.00%) GS8: 2 (14.29%) GS9: 2 (14.29%)
**CLINICAL STAGE**	pT2a: 5 (10.42%) pT2b: 20 (41.67%) pT2c: 1 (2.08%) pT3a: 15 (31.25%) pT3b: 6 (12.5%) pT4: 1 (2.08%)		pT2b: 6 (42.87%) pT3: 1 (7.14%) pT3a: 5 (35.71%) pT3b: 1 (7.14%) pT4: 1 (7.14%)
**Other Pathologies**			
Yes	Bladder tumour: 1 (2.08%) Colon Adenocarcinoma: 1 (2.08%)	Papillary carcinoma: 1 (12.50%) Urothelial carcinoma: 4 (50.00%) Bladder carcinoma: 3 (37.50%)	_
No	46 (95.84%)	_	14 (100%)
**Relapse**		_	
Yes	17 (35.41%)		6 (42.86%)
	GS5 (*n* = 2)		
	GS6 (*n* = 4)		
	GS7 (*n* = 6)		
	GS8 (*n* = 3)		
	GS9 (*n* = 2)		
No	29 (60.42%)		7 (50%)
	GS5 (*n* = 8)		
	GS6 (*n* = 5)		
	GS7 (*n* = 9)		
	GS8 (*n* = 4)		
Unknown	2 (4.17%)		1 (7.14%)
	GS7 (*n* = 1)		
	GS9 (*n* = 1)		
**Survival**			
Alive	2 (4.17%)		1 (7.14%)
	GS7 (*n* = 2)		
Alive with Disease	7 (14.58%)		2 (14.29%)
	GS5 (*n* = 1)		
	GS6 (*n* = 3)		
	GS7 (*n* = 2)		
	GS8 (*n* = 1)		
Alive without Disease	19 (39.58%)		6 (42.86%)
	GS5 (*n* = 5)		
	GS6 (*n* = 5)		
	GS7 (*n* = 4)		
	GS8 (*n* = 2)		
	GS9 (*n* = 3)		
Death unknown Cause	3 (6.25%)		1 (7.14%)
	GS7 (*n* = 1)		
	GS8 (*n* = 2)		
Death with Disease	10 (20.83%)		3 (21.43%)
	GS7 (*n* = 6)		
	GS8 (*n* = 2)		
	GS9 (*n* = 2)		
Death without Disease	5 (10.42%)		0
	GS5 (*n* = 4)		
	GS6 (*n* = 1)		
Unknown	2 (4.17%)		1 (7.14%)
	GS7 (*n* = 1)		
	GS9 (*n* = 1)		

Galectin-3 protein expression was evaluated in tissue slides containing normal prostate tissues from patients without PCa (tumour-free prostates) (*n* = 8), adjacent-normal prostate (only normal tissue from patients diagnosed with PCa) (*n* = 14) and tumour prostate tissues from PCa patients (*n* = 48). This table contains the clinical data of the patients enrolled in this study, including survival and relapse data. Stage pT2a refers to a tumour in one half or less of one side of the prostate, stage pT2b refers to tumour localized in more than one half of one side of the prostate, and stage pT2c refers to tumour involving both lobes of the prostate. Stages pT3a and pT3b mean that the tumour has spread beyond the prostate capsule or invaded the seminal vesicle(s), and y and at pT4 mean that the tumour has spread to other body organs. Abbreviations: GS: Gleason Score; PCa: prostate cancer.

## Data Availability

The data presented in this study are available on request from the corresponding author.
